# Malaria Frontline Project: strategic approaches to improve malaria control program leveraging experiences from Kano and Zamfara States, Nigeria, 2016–2019

**DOI:** 10.1186/s12913-023-09143-x

**Published:** 2023-02-11

**Authors:** Adefisoye Adewole, Olufemi Ajumobi, Ndadilnasiya Waziri, Amina Abdullahi Umar, Usaini Bala, Saheed Gidado, Gideon Ugbenyo, Edwin Simple, Isaac Igbaver, Adam Attahiru, Charles A. Michael, Belinda Uba, Patrick Nguku, Perpetua Uhomoibhi, Basheer Muhammad, Munira Ismael, Shelby Cash, John Williamson, Peter McElroy, Stephen Patrick Kachur, Kwame Asamoa

**Affiliations:** 1 African Field Epidemiology Network, Nigeria Country Office, Abuja, Nigeria; 2 School of Public Health, University of Nevada, Reno, Reno, Nevada USA; 3 National Malaria Elimination Program, Federal Ministry of Health, Abuja, Nigeria; 4 Kano State Ministry of Health, State Malaria Elimination Program, Kano, Nigeria; 5 Zamfara State Ministry of Health, State Malaria Elimination Program, Gusau, Nigeria; 6 Malaria Branch, Division of Parasitic Diseases and Malaria, Center for Global Health, Centers for Disease Control and Prevention, Atlanta, Georgia USA; 7 Mailman School of Public Health, Columbia University Medical Center, New York, NY USA

**Keywords:** Malaria Frontline Project, Healthcare workers, Capacity building, Malaria case management, Kano, Zamfara

## Abstract

**Background:**

The Malaria Frontline Project (MFP) supported the National Malaria Elimination Program for effective program implementation in the high malaria-burden states of Kano and Zamfara adapting the National Stop Transmission of Polio (NSTOP) program elimination strategies.

**Project implementation:**

The MFP was implemented in 34 LGAs in the two states (20 out of 44 in Kano and all 14 in Zamfara). MFP developed training materials and job aids tailored to expected service delivery for primary and district health facilities and strengthened supportive supervision. Pre- and post-implementation assessments of intervention impacts were conducted in both states.

**Results:**

A total of 158 (Kano:83; Zamfara:75) and 180 (Kano:100; Zamfara:80) healthcare workers (HCWs), were interviewed for pre-and post-implementation assessments, respectively. The proportions of HCWs with correct knowledge on diagnostic criteria were Kano: 97.5% to 92.0% and Zamfara: 94.7% to 98.8%; and knowledge of recommended first line treatment of uncomplicated malaria were Kano: 68.7% to 76.0% and Zamfara: 69.3% to 65.0%. The proportion of HCWs who adhered to national guidelines for malaria diagnosis and treatment increased in both states (Kano: 36.1% to 73.0%; Zamfara: 39.2% to 67.5%) and HCW knowledge to confirm malaria diagnosis slightly decreased in Kano State but increased in Zamfara State (Kano: 97.5% to 92.0%; Zamfara: 94.8% to 98.8%). HCWs knowledge of correct IPTp drug increased in both states (Kano: 81.9% to 94.0%; Zamfara: 85.3% to 97.5%).

**Conclusion:**

MFP was successfully implemented using tailored training materials, job aids, supportive supervision, and data use. The project strategy can likely be adapted to improve the effectiveness of malaria program implementation in other Nigerian states, and other malaria endemic countries.

**Supplementary Information:**

The online version contains supplementary material available at 10.1186/s12913-023-09143-x.

## Background

Malaria, a preventable and treatable disease, is a major cause of morbidity and mortality globally. An estimated 241 million cases of malaria occurred worldwide in 2020, of which 228 (95%) million cases were from the African region. African children under 5 years of age are the most vulnerable group accounting for 77% of all malaria deaths worldwide [1].

Nigeria has year-round malaria transmission, with 97% of its population at risk of malaria infection, transmission in the northern part of the country is however highly seasonal [2]. According to the World Health Organization’s (WHO) 2021 malaria report, Nigeria contributed 27% of the 241 million global malaria cases [1]. In the 2015 Nigeria Malaria Indicator Survey, 60% of outpatient visits to health facilities, 30% of childhood deaths, and 25% of infant mortality are attributed to malaria [3]. Under-five children experience an average of 2–4 episodes per year and account for as much as 90% of national malaria mortality with 36% attributable to malaria [4]. Nigeria’s high under-five malaria mortality is largely attributable to a health financing system that leaves many individuals uninsured, resulting in high out-of-pocket (OOP) medical expenditure that discourages care-seeking behaviour. The country accounts for the lowest rates of care-seeking for suspected cases of under-five malaria in the world accounting for less than 20% of all under-fives with fever being brought to health facilities for clinical consultation and parasitological testing [4]. Furthermore, malaria has been shown to account for over 40% of the total monthly curative healthcare costs incurred by households in Nigeria as compared to a combination of other illnesses; the cost of treating malaria and other illnesses depleted 7.03% of the monthly average household income, and treatment of malaria cases alone contributed 2.91% of these costs [5].

In Nigeria, the burden of malaria varies by geopolitical zones and states, with the northwestern states having the highest burden [6, 7]. The 2015 National Malaria Indicator Survey (MIS) showed the national average of malaria parasitemia by rapid diagnostic test (RDT) for children under five years old was 45%, but Kano and Zamfara States had parasitemia of 60.2% and 69.9% respectively [6].

The National Malaria Elimination Program (NMEP) implemented the National Malaria Strategic Plan 2014–2020 with [3] outlined targets to ensure transition from malaria control to malaria elimination.. The plan has multiple interventions based on WHO recommendations that are being scaled up for population impact. The interventions are Indoor Residual Spraying (IRS) across some geographical regions in the northern part of the country, universal coverage of Long Lasting Insecticidal Nets (LLIN), strategic use of larval source management (larvicide and environmental management), increased coverage of Intermittent Preventive Treatment (IPTp) with sulphadoxine-pyrimethamine (SP) for malaria prevention during pregnancy, strategic deployment of seasonal malaria chemoprevention in the northern part of the country (an area of high seasonal malaria transmission), and provision of prompt access to effective malaria case management with emphasis on parasitological confirmation before treatment [3]. Despite concerted efforts in implementing these interventions, malaria has continued to endanger lives, with resultant increase in morbidity and mortality amongst the population and socio-economic impact [3, 8].

In July 2012, the U.S. Centers for Disease Control and Prevention (CDC) and the African Field Epidemiology Network (AFENET) established the National Stop Transmission of Polio program (NSTOP) to strengthen the Nigeria Polio Eradication Program at the subnational (state and LGA) levels and strengthen the routine immunization program [9]. NSTOP staff provide technical support to implement the National Polio Eradication Emergency Plan in polio high-risk areas, strengthen routine immunization, and improve maternal and child health indices [9, 10]. NSTOP staff also respond to communicable diseases outbreaks like measles and rubella, across states in Nigeria.

**Fig. 1:**
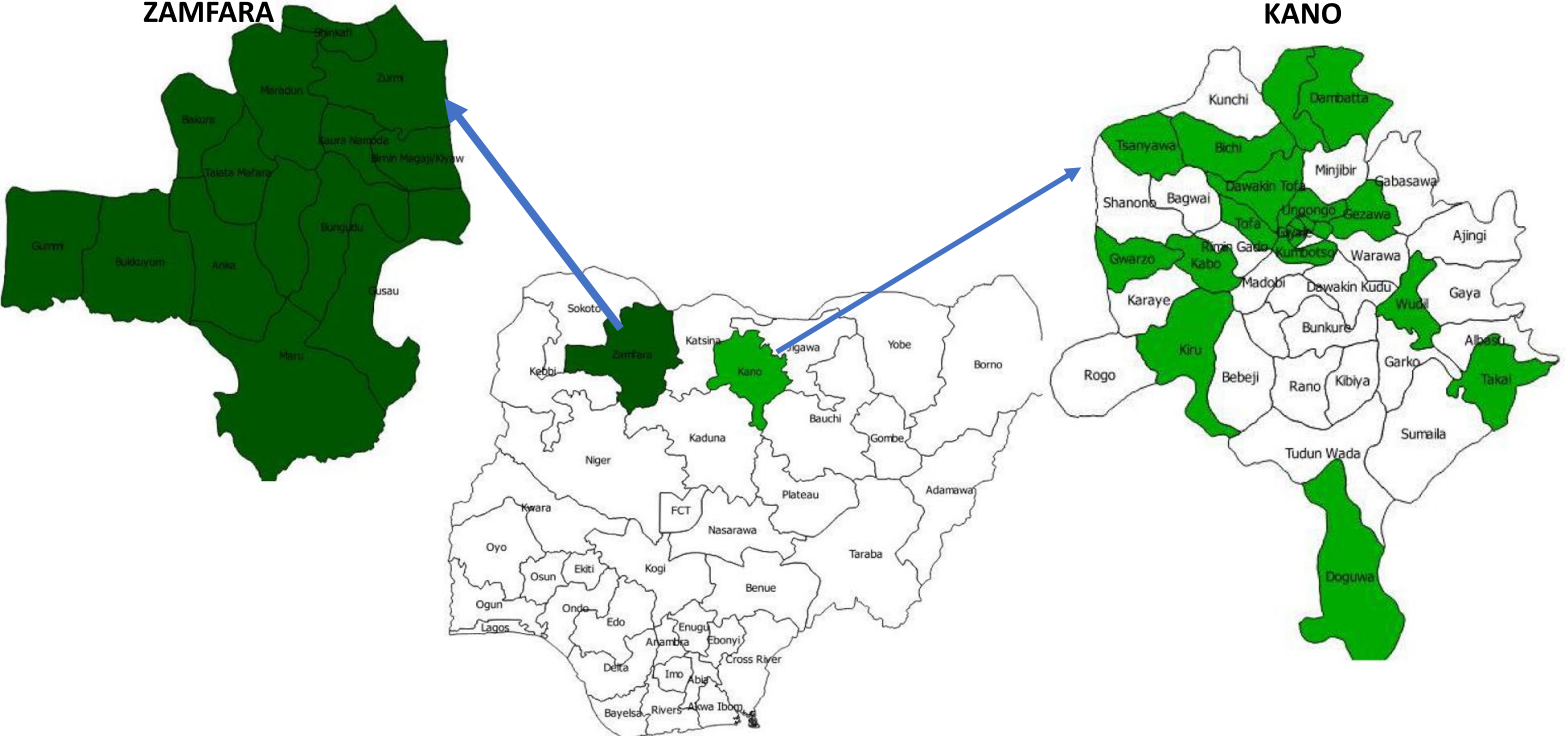
Map of Nigeria showing project LGAs in Kano and Zamfara States

In 2016, the CDC established the Malaria Frontline Project (MFP) in collaboration with AFENET/NSTOP and NMEP to support the elimination of malaria in Nigeria using the NSTOP structure. The project was implemented in the high malaria burden states of Kano and Zamfara. These states (Fig. 1) were selected because of their high malaria burden as documented in the 2015 MIS and their state governments’ commitment to their malaria elimination programs.

The objectives of the project were to strengthen the technical capacity of healthcare workers (HCWs) to implement malaria interventions (diagnosis, treatment, and prevention services), strengthen malaria surveillance and facilitate evidence-based decision making, and improve malaria commodities distribution to health facilities using routine health facility data. This paper describes MFP implementation and its contributions to malaria control programs in health facilities in Kano and Zamfara States, Nigeria.

### Project areas

**Fig. 2:**
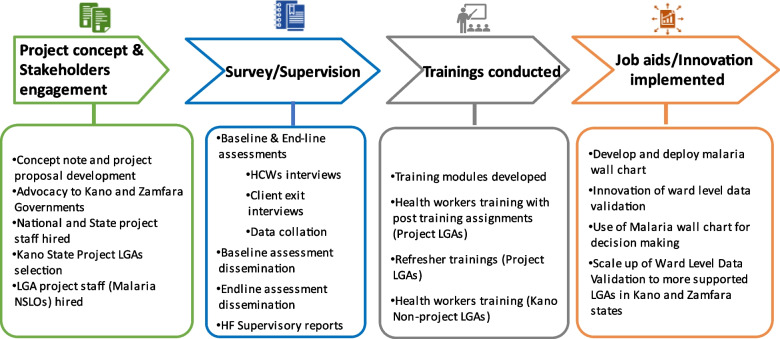
Overview of the Malaria Frontline Project Implementation in Kano and Zamfara States (2016–2019)

The project states are in the northwest geopolitical zone of Nigeria. Kano state has 44 local government areas (LGAs) and 484 political wards, with 14 LGAs and 147 wards in Zamfara state. The projected 2016 population for Kano and Zamfara states are 13,076,892 and 4,515,427 respectively [11]. MFP was implemented in 20 high-burden malaria LGAs out of the 44 LGAs in Kano state, and the entire 14 LGAs in Zamfara state (Fig. 1). In Kano state, the total number of health facilities in the project area was 726, of which 575 are publicly owned and 151 privately owned. Primary Health Care (PHC) facilities are 608 (83.7%), secondary facilities are 116 (16.0%) and tertiary facilities are two (0.3%). Zamfara state has 739 health facilities of which 713 are publicly owned and 26 are private. There are 715 (96.8%) PHCs, 22 (3.0%) secondary facilities and two (0.3%) are tertiary [12]. Pre-implementation and implementation activities of the Malaria Frontline Project (Fig. 2).

### Baseline project assessment

A baseline assessment was conducted (July – August 2016) before commencing MFP implementation. A modified President’s Malaria Initiative protocol for malaria intervention assessment in Nigeria was the basis for our assessments [13]. The baseline assessment data were obtained from (1) Interviews of primary and secondary facilities’ healthcare workers about their knowledge and practices of malaria case management and (2) exit interviews of health facility clients assessing malaria case management, LLIN distribution, and IPTp practices. Twenty-three PHCs from the project LGAs and 29 General Hospitals (GHs) in Kano State, and 23 PHCs and 16 GHs in Zamfara State were surveyed during the baseline assessment.

### Healthcare workers interviews

Two HCWs at each selected PHC (officer-in-charge and staff attending to patients) and at secondary health facilities, also known as General Hospitals (head of the out-patient department and staff attending to patients) were interviewed using a structured questionnaire. If the heads of the out-patient department do not consult with patients, then two HCWs attending to patients were interviewed about knowledge and practices regarding malaria case management.

### Client exit interviews

Client exit interviews (5 clients in each selected PHC facility) were conducted using questionnaires to assess client responses regarding HCWs’ adherence to national malaria case management guidelines, including both clinical and parasitological diagnostic confirmation of malaria and treatment with Artemisinin-based combination therapy (ACT). Interviewees were clients who came to the facility due to fever. If the client with fever was a child, the interview was conducted with the adult caregiver who brought the child to the facility. After interviewing a client, the next client to exit the PHC with fever as the main complaint was selected and interviewed until a total of 5 clients were interviewed during the facility visit.

Pregnant women in their second and third trimesters exiting the facility after antenatal care service were interviewed about receiving SP and the adherence of HCWs to the guidelines of SP administration under directly observed therapy. A total of five pregnant women were interviewed consecutively at each facility. 

Details of the methodology and findings of the baseline assessment is as referenced [14].

### Project implementation approach

**Fig. 3:**
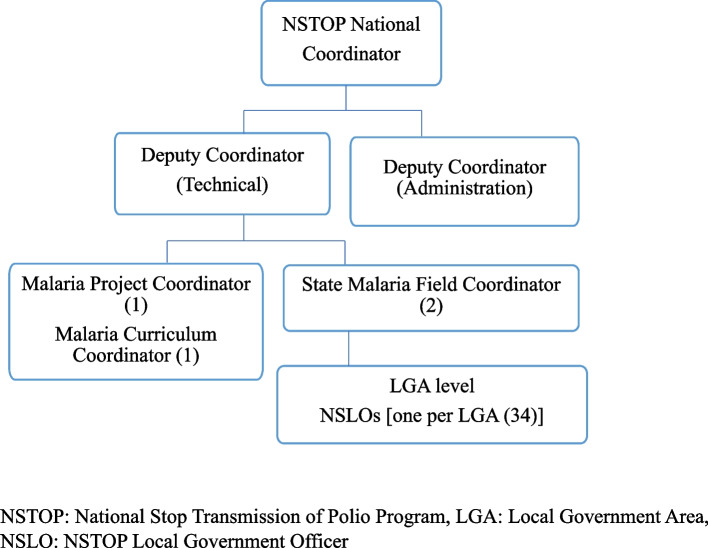
The Malaria Frontline Project Organogram

The MFP worked within the organogram of NSTOP at the national and sub-national levels, with two staff at headquarters, one at each state program, and one at each LGA totaling 38 MFP staff (Fig. 3). The project was modelled after the NSTOP implementation strategy. It centered on health system strengthening through capacity building of the health workforce at all levels. Assessments were conducted in the project states to collect baseline and end-line information of the state malaria control activities regarding case management, surveillance, malaria in pregnancy, and program management, with focus on leadership and coordination activities. Findings from the baseline assessment guided the MFP to adapt the NSTOP three-pronged training strategy. The three-prong strategy included classroom didactic training taught using structured lessons, post-training group work assignments with case studies, and health facility supportive supervisory visits conducting on-the-job training for challenged facilities. During the field practicum, trainees were given assignments to report back for the next training session one to two months later.

### Training strategy

Training materials were developed for different malaria control themes based on the findings from the baseline assessment. Training content was oriented towards the expected services to be delivered by HCWs at PHC or hospital at ward or LGA level respectively. The training used cascade approach based on the levels of healthcare administration in the country. The project’s training strategy comprises a didactic and post-training assignment (field work) components. A National training of trainers was conducted in Abuja, FCT for state level officers for each thematic modules, this was followed up with a state level training in Kano and Zamfara state for LGA level officers based on the thematic areas of interest facilitated by trained state level officers. A cascade level training for healthcare workers at the health facility level facilitated by trained LGA level officers with a post-training assignment as field work over some weeks which will later be assessed during the conduct of a new thematic modular training. The categories of trainees for the various thematic areas clearly stated. (Tables 1 and 2).

**Table 1:** Capacity building of healthcare workers in Kano State

Type of training	2017		2018		2019				Categories of Trainees
	**New Training**		**Refresher Training**		**New Training**		**Refresher Training**		
	**(20 LGAs)**		**(20 LGAs)**		**(24 LGAs)**		**(20 LGAs)**		
	**State**	**Cascade**	**State**	**Cascade**	**State**	**Cascade**	**State**	**Cascade**	
Overview of malaria	75	N/A	N/A	N/A	N/A	N/A	N/A	N/A	PHC coordinators, Malaria LGA officers/NSLOs
Case management	44	923	40	1278	44	1186	37	926	Malaria LGA officers/ NSLOs, HCWs
Malaria in pregnancy	71	690	60	1118	76	792	57	467	Maternal and child health officers, Malaria LGA officers/ NSLOs, HCWs
Surveillance	64	1034	112	986	83	1162	89	1016	M&E officers, Malaria LGA officers/NSLOs, HCWs
Data management DHIS2	101	N/A	N/A	N/A	51	N/A	57	N/A	LGA M&E officers, LGA Asst. M&E officers, Zonal M&E officers, Malaria LGA officers/NSLOs

**Table 2:** Capacity building of healthcare workers in Zamfara State

Type of training	2017		2018		2019		Categories of Trainees
	**New Training**		**Refresher Training**		**Refresher Training**		
	**State**	**Cascade**	**State**	**Cascade**	**State**	**Cascade**	
Overview of malaria	54	N/A	N/A	N/A	N/A	N/A	PHC coordinators, Malaria LGA officers/NSLOs
Case management	38	1013	28	1314	28	975	Malaria LGA officers/ NSLOs, HCWs
Malaria in pregnancy	60	336	42	483	42	426	Maternal and child health officers, Malaria LGA officers/ NSLOs, HCWs
Surveillance	50	938	88	1268	56	1222	M&E officers, Malaria LGA officers/NSLOs, HCWs
Data management DHIS2	85	N/A	N/A	N/A	46	N/A	LGA M&E officers, LGA Asst. M&E officers, Zonal M&E officers, Malaria LGA officers/NSLOs

Administratively, a state consists of LGAs and an LGA consists of wards, PHCs are situated in wards. Cascade training approach is a step-down training by a level to the next level below like national to state, state to LGA, LGA to ward and ward to the health facility level. Facilitators from a higher-level train the staff of the level next below them. To minimize interruption of service delivery, PHC training materials are adapted for one-day training with tailored must-know content essential for optimal health service delivery. The master trainers deliver the PHC level modular training to health workers in clusters at the ward level.

NMEP staff served as facilitators. An in-class didactic training was organized for the LGA health team and MFP NSTOP LGA officers (NSLOs), who were given post-training field assignments of four-to-six weeks duration to facilitate practical application of the classroom learning. Supportive supervision for health facility staff from LGA to facility levels was strengthened in collaboration with the state Malaria Elimination Programs and LGA team. The health authorities provided logistic support during the project that enabled the supervisory team to conduct more scheduled supportive visits than previously, as state and LGA activities permitted. During the visits, on-the-job training and mentoring for the facility staff was conducted where necessary if knowledge deficits were identified. An important component of the MFP strategy was the paired training of NSLOs with LGA health teams (especially the LGA malaria focal person) and the combined field visits. The paired training provided opportunities for the exchange of ideas and transfer of skills between the two groups and improvement in work relationships. Thematic trainings began in October 2016, and field implementation of the project at the LGA level commenced in January 2017. Supportive supervision with an updated supervision checklist commenced in April 2017.

### Training modules

Five modular training curricula were developed and implemented with NSLOs over a period of 36 months. The first training module focused on an “overview of malaria”. Because the NSLOs did not necessarily have health backgrounds, it was important to orientate them on malaria as a public health problem and introduce the NSLOs to the operation of the malaria program within the state.

To help strengthen health facility and LGA technical capacity, additional thematic training modules were developed on 2) malaria diagnosis and treatment, 3) prevention and treatment of malaria in pregnancy, 4) malaria surveillance, and 5) data management and analysis for health information systems. Refresher courses on these themes were organized at different times during the project implementation. Trainings, refresher trainings, and the number of persons trained are reflected in Tables 1 and 2.

Training and refresher training was conducted across the project (20) LGAs in Kano State, and 14 LGAs in Zamfara state with cascade level trainings done. LGA level officers (PHC Coordinators, Malaria LGA officers, M&E officers, Maternal and Child Health officers, and Malaria NSLOs) were participants in the state level training. Trained LGA level officers later then conduct a 1-day cascade level training to health facility officers. In 2019, Kano State health authorities requested for the expansion of the project to all LGAs in the state so further trainings were conducted for the remaining 24 LGAs. (Table 1).

### Case management and malaria in pregnancy

Training materials for malaria case management and malaria in pregnancy were based on the national guidelines tailored to the HCWs expected service provision at the PHC and secondary health facility levels. Job aids for the diagnosis and treatment of uncomplicated malaria and the prevention of malaria in pregnancy algorithm, including IPTp, were developed and distributed to all health facilities in the project states to routinely refresh HCW knowledge.

### Surveillance and data analysis for decision making

Health facility data are recorded routinely using the national health management information system (NHMIS) forms and registers. Data from the health facilities are transferred to the monthly summary form (MSF) and submitted to the Monitoring and Evaluation officer of the LGA. This officer at the LGA collates the facility MSF and enter the data into the District Health Information System version 2 (DHIS2) platform. The baseline assessment conducted earlier in the project revealed major gaps in surveillance and data management with missing data, incorrect data summation, and lack of data analysis at facility and LGA levels. These findings informed the development of surveillance-specific training module for malaria data analysis and surveillance training of LGA and health facility staff.

A minimum number of malaria indicators for monitoring and comparison of program performance at the LGA, ward, and facility level were selected in collaboration with health authorities. The following indicators were selected at the facility level: outpatient febrile cases, febrile cases tested for malaria, test-positive cases given correct antimalaria treatment, antenatal care (ANC) attendees due for IPTp who were given SP, and pregnant women and children under five years old issued an LLIN. The training focused on the interpretation of data analyses, thereby facilitating the use of data for decision making and helping to rectify problems at health facilities. During the project implementation, efforts were made to enhance the visibility of malaria data and increase its use to improve program performance. A Malaria Monitoring wall chart (Fig. 3) was developed and distributed to all health facilities, ward and LGA offices in the project states. Key elements of the wall chart summarizing monthly data were total outpatient visits, total ANC visits, and malaria in pregnancy, and LLIN uptake among children under five years and pregnant women. Key indicators on the wall chart were monitored for the purpose of initiating public health action. The chart helped users understand the malaria trends, adherence/non-adherence to national diagnosis and treatment guidelines, uptake of intermittent preventive treatment during pregnancy, and LLIN uptake among pregnant women and children under five years. Gaps such as transcriptional, summarizing, and trans-positional errors were identified by HCWs at the facility and resolved before data entry into DHIS by LGA Monitoring and Evaluation Officer.

### End-line project assessment

The end-line assessment was conducted after three-years of the project implementation (October – December 2019) using the same protocol for the baseline assessment [13, 14]. The baseline and end-line assessments used the same protocol to enable comparison of the findings except replacing LGAs not accessible due to security issues. During the end-line assessment, some health facilities that were not visited in both project states due to non-functionality and security concerns at the time of the baseline assessment were replaced and surveyed. A total of 24 PHCs and 36 GHs in Kano State, and 24 PHCs and 16 GHs in Zamfara State were surveyed during the end-line assessment. Supervisory checklist being filled by NSLOs during health facility supportive supervision provide information on proportion of health facilities giving ACT to malaria negative patients, pregnant women who received IPTp, stockout of SP, and use of analyzed data from malaria monitoring wall chart for decision making.

### Data analysis

Descriptive analyses of frequencies and corresponding percentages of the baseline and end-line assessments were conducted for the health facility components. The merged dataset was analyzed using Statistical Package for Social Sciences (SPSS) version 23 and Statistical Analysis System (SAS) version 9.4. Trends of selected malaria indicators from 2017 to 2019 from supervisory reports are described.

## Results

### Healthcare workers interviews

A total of 158 (Kano: 83, Zamfara: 75) and 180 (Kano: 100, Zamfara: 80) HCWs were interviewed during the baseline and end-line assessments, respectively. Fifty-six percent of HCWs interviewed in the baseline assessment and 59% of those interviewed in the end-line assessment work at PHCs.

#### Client exit interviews

A total of 131 (Kano: 82, Zamfara: 49) and 978 (Kano: 667, Zamfara: 311) clients were interviewed during the baseline and end-line assessments, respectively. At ANCs, five pregnant women were interviewed consecutively at each facility.

### HCWs’ knowledge of and adherence to national guidelines on malaria diagnosis and treatment

#### Knowledge

##### Correct diagnosis

Diagnostic criteria for malaria were defined as fever, other symptoms, and signs suggestive and parasitological confirmation of malaria.

Over 90% of the respondents in the baseline and end-line assessments knew the correct diagnostic criteria for malaria (Kano: 94.0% to 92.0%, respectively; Zamfara: 94.7% to 98.8%, respectively). In Kano State, the proportion of HCWs who responded that clinical diagnosis only was the diagnostic criteria of malaria increased in the end-line assessment (Kano: 2.5% to 8.0%) but in Zamfara State there was a decline of the proportion of HCWs who did not know the correct diagnostic criteria (Zamfara: 4.1% to 1.3%) (Table 3).

**Table 3:** Healthcare worker’s knowledge of national guidelines on diagnosis and treatment of malaria in Kano and Zamfara

		**Zamfara**		**Kano**	
**Characteristics**		**Baseline** ***N* = 75** **n (%)**	**End-line** ***N* = 80** **n (%)**	**Baseline** ***N* = 83** **n (%)**	**End-line** ***N* = 100** **n (%)**
**Knowledge on diagnostic criteria of malaria**	Clinical diagnosis	3 (4.1)	1 (1.3)	2 (2.5)	8 (8.0)
	Clinical plus parasitological diagnosis	71 (95.9)	79 (98.8)	78 (97.5)	92 (92.0)
**Knowledge on recommended first line treatment for uncomplicated malaria**	Artemether-Lumefantrine or Artesunate-Amodiaquine	52 (69.3)	52 (65.0)	57 (68.7)	76 (76.0)

##### Treatment

Over 60% of respondents correctly identified Artemether-Lumefantrine as the first-line drug for uncomplicated malaria treatment at baseline and end-line (Kano: 68.7% to 76.0%, respectively; Zamfara: 69.3% to 65.0%, respectively) (Table 3).

##### HCW-reported adherence to guidelines

The proportion of HCWs who responded that they adhere to the national guidelines of clinical plus parasitological confirmation for malaria diagnosis and treatment with ACT showed improvement from baseline to end-line in both states (Kano: 36.1% to 73.0%; Zamfara: 39.2% to 67.5%) (Table 4).

**Table 4:** Healthcare worker’s adherence to guidelines on malaria diagnosis and treatment practices in Kano and Zamfara

		**Zamfara**		**Kano**	
**Characteristics**		**Baseline** ***N* = 75** **n (%)**	**End-line** ***N* = 80** **n (%)**	**Baseline** ***N* = 83** **n (%)**	**End-line** ***N=* 100** **n (%)**
**Adherence to national diagnosis and treatment guidelines**		29 (39.2)	54 (67.5)	30 (36.1)	73 (73.0)
**Malaria diagnostic practices**	*Confirmation based on diagnostic tests	71 (94.7)	80 (100.0)	80 (96.4)	100 (100.0)
Malaria treatment practices for uncomplicated malaria (medicines)**	Correct	74 (98.7)	78 (97.5)	79 (95.2)	96 (96.0)

^**^ Correct response is artemisinin-based combination therapy; other responses are incorrect (oral artesunate, quinine tablet, quinine injectables, sulphadoxine-pyrimethamine, chloroquine tablet, chloroquine injection, herbal preparations, and paracetamol)

^*^Diagnostic tests refers to both rapid diagnostic test and microscopy

The proportion of HCWs who reported they adhere to parasitological diagnosis of malaria (using RDT) in both states was high at baseline and increased at the end-line assessment (Kano: 96.4% to 100.0%; Zamfara: 94.7% to 100.0%).

The proportion of HCWs who used the recommended ACTs medicines for treatment of uncomplicated malaria was high between baseline and end-line (Kano: 95.2% to 96.0%; Zamfara: 98.7% to 97.5%) (Table 4).

Facilities supervisory visit reports from 2017–2019 showed declines in the proportions of health facilities that use ACT for treatment of malaria-negative patients (Kano: 2.6%, 1.5% and 1.1%; Zamfara: 1.9%, 0.5%, and 0.5%).

### HCWs’ knowledge of Malaria treatment during pregnancy

Between baseline and end-line assessments, the proportion of HCWs with correct knowledge about treatment of uncomplicated malaria in the 1^st^ trimester of pregnancy with quinine tablets according to the national guidelines increased in Kano (36.1% to 54.0%) but decreased slightly in Zamfara (25.3% to 23.8%). The proportion of HCWs with correct knowledge for treatment of uncomplicated malaria in 2^nd^ and 3^rd^ trimester with ACT increase in Kano state but less so in Zamfara state (Kano: 56.6% to 69.0%; Zamfara: 60.0% to 61.3%) (Table 5).

**Table 5:** Healthcare worker’s knowledge of intermittent preventive treatment and malaria in pregnancy in Kano and Zamfara

		**Zamfara**		**Kano**	
**Characteristics**		**Baseline** ***N* = 75** **n (%)**	**End-line** ***N* = 80** **n (%)**	**Baseline** ***N* = 83** **n (%)**	**End-line** ***N* = 100** **n (%)**
**Knowledge of drugs for IPTp**	Sulphadoxine-pyrimethamine (correct)	64 (85.3)	78 (97.5)	68 (82.0)	94 (94.0)
	Non-Sulphadoxine-pyrimethamine (incorrect)	11 (14.7)	2 (2.5)	15 (18.0)	6 (6.0)
Knowledge of when to commence IPTp*	After 13 weeks or at quickening (16 weeks) – correct	59 (78.7)	64 (82.1)	75 (90.0)	85 (85.0)
	Before 13 weeks or after quickening (16 weeks)—incorrect	16 (21.3)	14 (17.9)	8 (10.0)	15 (15.0)
**Drug given for treatment of uncomplicated malaria during 1**^**st**^** trimester of pregnancy**	Quinine tablets	19 (25.3)	19 (23.8)	30 (36.1)	54 (54.0)
	Non-Quinine tables	56 (74.7)	61 (76.3)	53 (63.9)	46 (46.0)
**Drug given for treatment of uncomplicated malaria during 2**^**nd**^** and 3**^**rd**^** trimesters of pregnancy**	ACT	45 (60.0)	49 (61.3)	47 (56.6)	69 (69.0)
	Non-ACT	30 (40.0)	31 (38.8)	36 (43.4)	31 (31.0)

### HCWs’ knowledge and practice of prevention of malaria during pregnancy

#### IPTp

There was an increase in the proportions of HCWs who knew the recommended medicine for IPTp in Kano (82.0% to 94.0%), and in Zamfara (85.3% to 97.5%). The proportion of healthcare workers who knew the time to commence IPTp decreased in Kano from 90.0% to 85.0%, with an increase in Zamfara from 78.7% to 82.1%. (Table 5). However, the proportion of pregnant women receiving IPTp under direct observation therapy (DOT) at ANC increased [Kano: 42.9% (6/14) to 92.7% (114/131); Zamfara: 0.0% (0/6) to 77.5% (79/140)]. In Kano State, 12 of 14 (85.7%) pregnant women had received one or more doses of IPTp at baseline, and 121 of 131 (92.4%) had received one or more doses at end-line. In Zamfara State, only one of six (16.7%) of pregnant women had received one or more doses at the time of the baseline assessment, but at the time of the end-line assessment 85 of 105 (81.0%) had received one or more doses of IPTp. During the conduct of the baseline assessment, some health facilities could not be visited because of security challenges and inaccessible terrain during the rainy season. These contributed to the small sample size of the clients we interviewed. Also, for some health facilities, days selected for the baseline assessments did not coincide with ANC days in facilities visited, resulting in low client turn out (Table 6).

**Table 6:** Intermittent Preventive Treatment uptake among antenatal care clients interviewed in health facilities visited in Zamfara and Kano

	Zamfara		Kano	
Characteristics	**Baseline**^**a**^ ***N* = 6** **n** **(%)**	**End-line**^**b**^ ***N* = 140** **n** **(%)**	**Baseline** ***N* = 14** **n** **(%)**	**End-line** ***N* = 131** **n** **(%)**
**Received** **SP** **as** **directly** **observed** **therapy** **during** **current** **visit**	0 (0.0)	79 (77.5)	6 (42.9)	114 (92.7)
Number of doses				
None	4 (66.7)	20 (18.3)	2 (14.3)	10 (7.6)
One or more doses	1 (16.7)	89 (81.7)	12 (85.7)	121 (92.4)

*IPTp*  intermittent preventive treatment, *SP* sulphadoxine-pyrimethamine

^a^One of the pregnant women was not sure if she had SP or not

^b^31 pregnant women were not sure if they had SP or not

Reports from health facility supportive supervisory visits from 2017–2019 showed an increase in the proportion of health facilities that provided IPTp services as DOT [Kano: 83.3%, 97.9%, 97.3%; Zamfara: 83.5%, 88.1%, 88.6%].

#### LLINs

The percentage of pregnant women who were provided with LLINs at ANC visits was 7.1% at baseline and 38.7% at end-line in Kano State, and 33.3% at baseline and 23.3% at end-line in Zamfara State (Table 7).

**Table 7:** Healthcare worker’s advice on long-lasting insecticidal nets use and receipt among antenatal care clients interviewed in healthcare centers visited in Zamfara and Kano

	Zamfara PHCs		Kano PHCs	
Characteristics	**Baseline** ***N* = 6** **n** **(%)**	**End-line** ***N* = 129** **n** **(%)**	**Baseline** ***N* = 14** **n** **(%)**	**End-line** ***N* = 137** **n (%)**
Client advised on LLIN use	5 (83.3)	102 (79.1)	11 (78.6)	125 (91.2)
Clients received LLIN	2 (33.3)	30 (23.4)	1 (7.2)	111 (81.0)

### Client exit interviews: malaria testing

The proportion of febrile patients that clinicians requested malaria parasitological testing increased in both states (Kano:72.8% to 97.2%; Zamfara79.2% to 96.6%) and the proportions of patients tested were high in both states (Kano: 100% to 99.2%; Zamfara: 94.7% to 99.3%) (Table 8).

**Table 8:** Malaria testing and treatment among exit clients interviewed at primary health clinics in Zamfara and Kano

	Zamfara PHCs		Kano PHCs	
Characteristics	**Baseline** ***N* = 48** **n** **(%)**	**End-line** ***N* = 147** **n** **(%)**	**Baseline** ***N* = 81** **n** **(%)**	**End-line** ***N* = 145** **n (%)**
**Clinician request for malaria testing**	38 (79.2)	142 (96.6)	59 (72.8)	141 (97.2)
	***N* = 38**	***N* = 142**	***N* = 59**	***N* = 141**
**Client got tested**	36 (94.7)	141 (99.3)	59 (100.0)	140 (99.3)
	***N* = 30**	***N* = 138**	***N* = 54**	***N* = 131**
**Waiting time for test result ≤ 1 h**	29 (96.7)	138 (100.0)	51 (94.4)	119 (90.8)

### Health facility supervisory visit reports

**Fig. 4:**
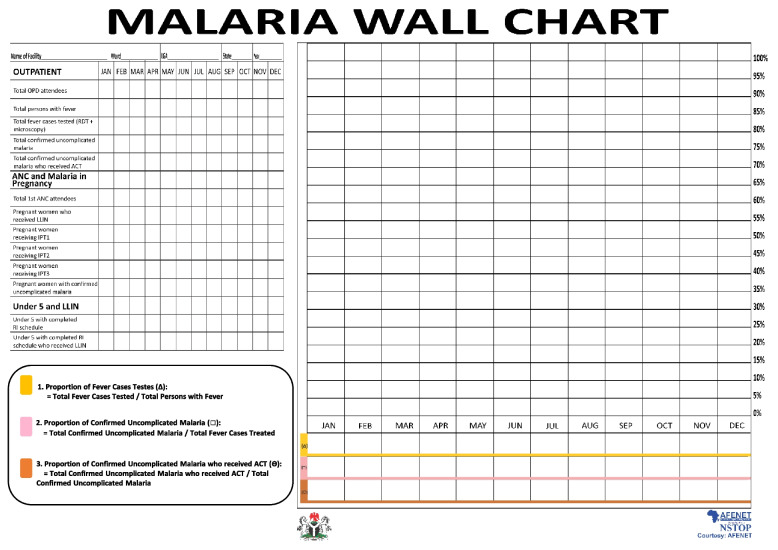
Malaria monitoring wall chart

**Fig. 5:**
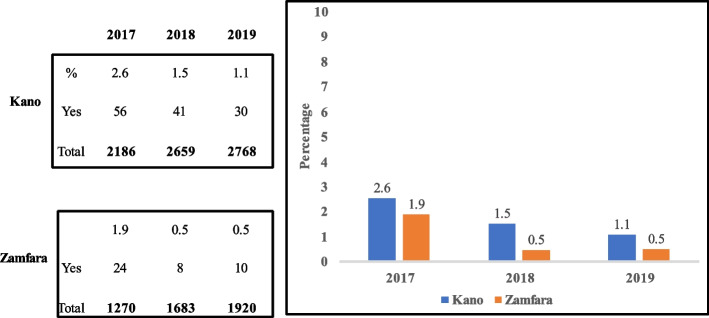
Proportion of health facilities that gave ACT to negative malaria patient

**Fig. 6:**
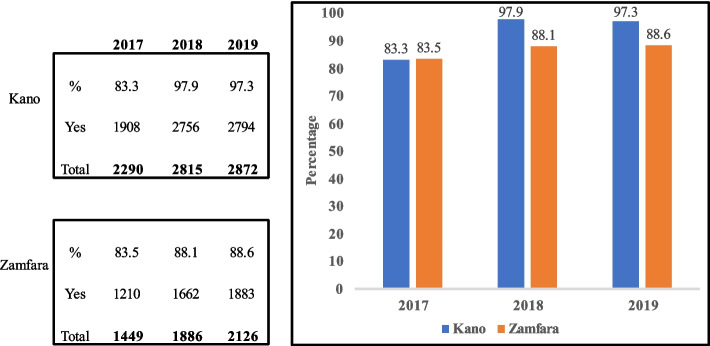
Healthcare worker observation of client take IPTp

**Fig. 7:**
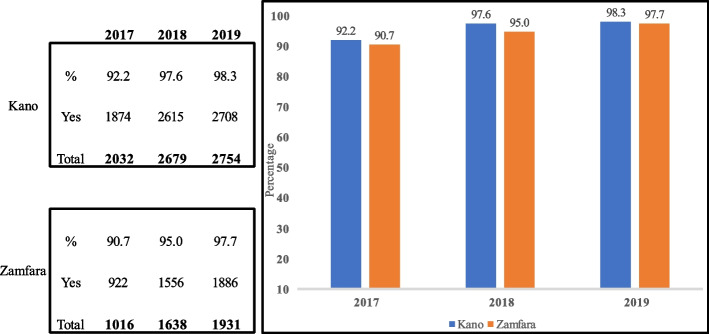
Proportion of health facilities using analyzed data for decision

Supervisory reports during the project period (2017–2019) based on the facility malaria monitoring wall chart (Fig. 4), showed unconfirmed febrile patient receiving ACT reduced [(Kano: 56 (2.6%), 41 (1.5%) and 30 (1.1%); Zamfara: 24 (1.9%), 8 (0.5%) and 10 (0.5%) (Fig. 5), IPTp for pregnant women given by direct observation of client (DOT) improved (Kano: 1908 (83.3%), 2756 (97.9%) and 2794 (97.3%); Zamfara: 1210 (83.5%), 1662 (88.1%) and 1883 (88.6%) (Fig. 6) and, the proportion of facilities using analyzed data for decision making also improved (Kano: 1874 (92.2%), 2615 (97.6%) and 2708 (98.3%); Zamfara: 922 (90.8%), 1556 (95.0%) and 1886 (97.7%) (Fig. 7).

## Discussion

The MFP leveraged the success of the NSTOP program strategy to build capacity of HCWs in malaria case management, surveillance, and data management using the three-prong training strategy approach of didactic training, post-training assignments, and health facility supportive supervisory visits with on-the-job training and mentorship [9]. The content of the training materials was very much oriented to the expected services that is to be delivered at the facility level. There were improvements in some of the malaria control interventions. Improved case management practices reduced clinically diagnosed malaria cases, increased testing of all febrile cases before treatment, and improved use of data for decision making at all levels. Gaps from the baseline assessment were addressed through targeted interventions of conduct of thematic modular trainings and health facility supportive supervisory visits during project implementation and measured through the end-line.

One of the objectives of MFP is capacity building of HCWs at LGA, ward and health facility levels. The National Malaria Strategic Plan 2014–2020 recommends that, by 2020, all febrile persons seeking care should be tested for malaria to confirm or rule out a parasitological diagnosis. The national malaria guideline states that all fever cases should be tested by rapid diagnostic test (RDT) or microscopy [15, 16]. Prior to the implementation of MFP many health workers practiced presumptive diagnosis of malaria, which was contrary to the national recommendations for management of uncomplicated malaria aimed at mitigating the problem of misdiagnosis leading to mismanagement of suspected malaria cases [17–19]. Parasitological diagnosis is a component of malaria case management. Improper use of ACTs without confirmatory diagnosis will result in negative clinical and economic impact [20].

The thematic modular trainings, post-training assignments on malaria case management practices, provision of hard copies of a simplified diagnosis and treatment algorithm in the consulting room, and regular (as permitted by other activities) LGA health team supportive supervisory visits mostly driven by NSLOs and on-the-job mentoring are all contributing factors to improvements in health workers knowledge and adherence to national guidelines on malaria diagnosis and treatment.

The capacity building of HCWs in filling out the malaria monitoring wall chart improved data capturing using the monthly summary form and increased health facilities using analyzed data for decision making. All health facilities implemented the use of monitoring wall chart successfully. This provided an opportunity to use data for decision making at the health facility level. But it also improved data validation at that level before the data were transferred into monthly summary form. Data validation meetings provided another layer of data quality assurance such as correction of identified errors for the data aggregated using the MSF. The gaps such as transcriptional, summarizing, and trans-positional errors were identified and resolved before data entry into DHIS. Impact of the project as reflected in key malaria indicators, include declines in clinically diagnosed malaria cases and health facilities administering ACT to negative malaria patients.

The improved adherence by HCWs to national guidelines on diagnosis and treatment of malaria is similar to studies conducted in other states in the north west region of the country, which found the expectation of the patient to be given antimalarial treatment and HCWs’ prior receipt of training on malaria case management (both by governmental and non-governmental organizations) were predictors of using rapid diagnostic test results to guide treatment [21, 22]. Studies have shown that when HCWs practice evidence-based medicine most especially, testing fever cases before treatment, resources are conserved, selective pressure on ACT is reduced, and ultimately potential early resistance to anti-malarial medicines can be averted [23]. The majority of HCWs at PHCs in the MFP states were community health extension workers (CHEWs). Within the context of PHC settings in Nigeria, CHEWs play a major role in the provision of basic health care services and have a higher likelihood of adherence to defined guidelines through periodic trainings and re-trainings, as conducted by MFP with support from project states malaria programs and regular health facility supportive supervision and on-the-job mentoring. The study findings of improved adherence to national guidelines on diagnosis and treatment is similar to other studies that reported the relationship between adherence to test results and training [24, 25]. There was improvement in HCW’s knowledge about drugs for intermittent preventive treatment during pregnancy (IPTp) using SP and, in Zamfara, about the time of commencement of IPTp. This was likely attributable to the development and distribution of a malaria-in-pregnancy algorithm as a job aid for frontline line workers providing ANC services in health facilities across the two project states. This finding is similar to a study conducted in south-west Nigeria [26] and in contrast to other studies [27, 28]. However, the coverage of IPTp was very low, not meeting the 2016 national target of 80%. This low coverage of IPTp is similar to the study conducted in south-west Nigeria in which late ANC bookings and stock out of sulphadoxine-pyrimethamine were associated with the low IPTp uptake [29]. Another study in Malawi found low IPTp uptake was associated with number of ANC visits [30] It is important to conduct a detailed study of factors contributing to the low uptake of IPTp in the second and third trimesters to improve the protection of the pregnant woman and her unborn child. The stockout of SP for IPTp during the MFP implementation contributed to the low uptake, hence partners and state health authorities need to procure adequate amount of SP and have equitable distribution to health facilities.

The client exit interview confirmed the increase in HCWs’ requests for parasitological confirmation of diagnosis and the increased prescription of ACT to treat malaria cases. This finding was similar to a study conducted in North-Central Nigeria in which healthcare providers prescribe ACTs based on test results, and good knowledge of malaria diagnosis and treatment as a result of prior trainings on malaria case management [31]. This finding was in contrast with a study conducted in Ebonyi State, Nigeria [32] in which health workers had poor perceptions of RDTs as reflected in the level of reported wrong prescription practices, and in contrast with other studies [33, 34].

Furthermore, the improvement in clinician’s adherence to national guidelines on diagnosis and treatment can be likely attributed to the collaborative efforts of the national and state malaria programs, MFP, and other malaria implementing partners. With continued trainings and re-trainings, clinicians now appreciate giving feedback to their clients on test results and diagnosis. However, the occasional shortages of diagnostic kits and antimalarial drugs during the project implementation affected HCWs adherence to national guidelines when faced with stockouts. It will be good for the state to assure adequate supply of antimalarial commodities to all health facilities because currently some facilities are not supported by any partner, and the state government is unable to provide adequate commodities. Despite facility records showing high percentages of antimalarial drugs given to confirmed malaria patients, the findings from client exit interview show a moderate result. This could be due to antimalarial commodities stock at the time of the assessment or the need to check on data quality at the facility level. Regular supportive supervision by LGA team to facilities is an important contributing factor for health workers adherence to the national guidelines.

The MFP implementation experienced some limitations which however were addressed. There was high transfer or movement of HCWs between states, within state, and rotation to different departments after training by MFP. This was however addressed by regular advocacy and follow up visits to the State Primary Health Care Board/Agency for retention of service providers across supported health facilities in both project states. Also, MFP did not provide any malaria commodities in the project area, however worked with other malaria implementing partners in ensuring continuous and uninterrupted supply of malaria related commodities to supported health facilities. Sustainability of innovative approaches implemented during the project implementation have been institutionalized with the state and LGA health teams’ involvement and ownership. The conduct of ward level data validation as a peer-to-peer capacity building platform for service providers with the routine health facility supportive supervision and mentorship, and use of data at the health facility for decision making have greatly helped resulted in data quality improvement during and after the project’s implementation in Kano and Zamfara states. The approach provided the opportunity to validate data efficiently in much smaller groups compared to the previous LGA level data validation and it facilitated earlier submission of the data into DHIS2. The ward level data validation meeting was adopted by NSTOP for routine immunization program. NMEP planned to expand the project to other states but there have been lot of changes in the program hierarchy. Our findings are not generalizable to other settings because of the non-probability approach to sampling the study population and the absence of control health facilities.

## Conclusion

MFP was successfully implemented in both Zamfara and Kano States using tailored training materials, job aids, and supportive supervision, contributing to improved HCWs knowledge and adherence to guidelines on malaria case management. The strategy used to implement this project can be adapted to improve the efficiency and effectiveness of malaria program implementation in other states in Nigeria as well as other malaria endemic countries, especially on the African continent. Building the capacity of health facility leads in basic data analyses and use of the data for management will improve the health delivery system at the lower levels of the health system.

State malaria programs should continue with political commitment for implementation of malaria interventions**,** continuous capacity building of HCWs on adherence to national guidelines**,** sustaining and improving health facility supportive supervisory visits**,** sustaining LGA monthly data analysis of key malaria indicators**,** and the production of malaria monitoring wall charts.

## Supplementary Information

**Additional file 1.**

## Data Availability

The datasets supporting the conclusions of this article are included within the article.

## Abbreviations

WHO: World Health Organization
NMEP: National Malaria Elimination Program
IRS: Indoor Residual Spraying
LLIN: Long Lasting Insecticidal Nets
IPTp: Intermittent Preventive Treatment during Pregnancy
SP: Sulphadoxine-pyrimethamine
MIS: Malaria Indicator Survey
RDT: Rapid diagnostic test
CDC: Centers for Disease Control and Prevention
MFP: Malaria Frontline Project
AFENET: African Field Epidemiology Network
NSTOP: National Stop Transmission of Polio Program
HCWs: Healthcare workers
LGAs: Local Government Areas
PHC: Primary Health Care
NSLOs: NSTOP Local Government Officers
NHMIS: National Health Management Information System
MSF: Monthly summary form
DHIS2: District Health Information System version 2
ANC: Antenatal care
GHs: General Hospitals
ACT: Artemisinin-based combination therapy
SPSS: Statistical Package for Social Sciences
SAS: Statistical Analysis System
DOT: Direct observation therapy
CHEWs: Community health extension workers
