# Supplementary material for: Malaria Frontline Project: strategic approaches to improve malaria control program leveraging experiences from Kano and Zamfara States, Nigeria, 2016–2019
Source: BMC Health Serv Res. 2023 Feb 11;23:147. doi: 10.1186/s12913-023-09143-x (PMC9922450; doi:10.1186/s12913-023-09143-x)
Supplement: Supplementary file 1 — Additional file 1. [file 12913_2023_9143_MOESM1_ESM.docx]

**QUESTIONNAIRE ON KNOWLEDGE AND PRACTICES OF HEALTHCARE WORKERS ON MALARIA PREVENTION STRATEGIES AND CASE MANAGEMENT IN KANO AND ZAMFARA STATES, NIGERIA**

Dear Respondents,

My name is ____________________________________. I am working with the National Malaria Elimination Program (NMEP). We are conducting malaria baseline/endline assessment including assessment of healthcare workers’ knowledge and practices on malaria. I would like to ask you some few questions on malaria. All information would be treated confidentially. Refusal to participate will not in any way affect your job as a healthcare provider in your facility.

Please kindly ensure that you answer all questions truthfully.

Thanks for your cooperation.

**Do you consent to participate?**

Yes No

Serial no: _____________ Date: ________________

Name of health facility: ____________________________________

Category of health facility: Primary ( ) Secondary ( ) Tertiary ( )

Local Government Area (LGA): ________________

State: ______________________

| RECORD THE TIME THE INTERVIEW STARTED | \|  \|  \|  \|  \| . \|  \|  \|  \|  \| \| --- \| --- \| --- \| --- \| --- \| --- \| --- \| --- \| --- \| \|  \|  \|  \|  \| . \|  \|  \|  \|  \| |
| --- | --- | --- | --- | --- | --- | --- | --- | --- | --- | --- | --- | --- | --- | --- | --- | --- | --- | --- | --- |

**SECTION A (Socio-demographic characteristics of respondents).**

1. How old were you on your last birthday (in years)? ____________________
2. What is your Ethnic group? (a) Yoruba (b) Ibo (c) Hausa (d) Others (Please Specify)
3. Highest level of Education: (a) Didn’t go to school/ (Remove) No formal education (b) Primary education only (c) Secondary education only (d) Tertiary education (e) Others, please specify……. (e.g., Koranic School)
4. What is your religion? (a) Christianity (b) Islam (c) Traditional (d) None (e) Others Please Specify…….
5. Marital Status: (a) Single (b) Married (c) Divorced (d) Separated (e) Co-habiting (f) Widowed
6. What is your current job cadre? (a) Medical Doctor (b) registered nurse/ midwife (c) Registered nurse (d) CHEW (e) JCHEW (f) CHO (g) Health Assistant (h) Pharmacy technician (i) Pharmacist (j) Laboratory technician (k) Laboratory scientist (l) record officer (m) Others, please specify………
7. How long have you been in health practice (in years)? ………….
8. How many patients do you attend to in a day? (a) 1-25 patients/day (b) 26-50 patients/day (c) >50 patients/ day

**SECTION B: TRAINING**

9. Please insert as appropriate

| **S/N** | **Type of training** | **Have you or anyone in your facility been trained on? Yes/No/Don’t know** | **Was this on-the-job training? Yes/No/Don’t know** | **Was this a formal classroom training? Yes/No/Don’t know** | **How long ago was most recent training? (a) < 1year (b) 1 – 2 years (c) 3 years or more** | **Who provided the training (a) federal government**  **(b) state government (c) health facility management (d) donor agencies/NGOs (e) others** |
| --- | --- | --- | --- | --- | --- | --- |
| 1 | Malaria case management (without RDT use) |  |  |  |  |  |
| 2 | Malaria case management (inclusive of RDT use) |  |  |  |  |  |
| 3 | Malaria in pregnancy |  |  |  |  |  |
| 4 | Malaria preventive services (orientation on net use and hanging) |  |  |  |  |  |
| 5 | Malaria preventive services (orientation on larval source management) |  |  |  |  |  |
| 6 | Malaria preventive services (orientation on indoor residual spraying) |  |  |  |  |  |
| 7 | *NHMIS tools |  |  |  |  |  |

*NHMIS: National Health Management Information System

**SECTION C:** **KNOWLEDGE OF HEALTH CARE PROVIDERS ON MALARIA PREVENTION STRATEGIES**

10. Are you aware of the national strategies and guidelines for prevention and control of malaria in pregnancy? 🞏 Yes 🞏 No

11. Malaria is **a** (tick the best option) (read out options): a) Protozoa disease b) bacterial c) Viral d) fungal e) Others, …. please specify

12. It is caused **by** (tick all that apply) (read out options): a) Mosquito b) Plasmodium spp. c) drinking impure water d) lack of environmental sanitation e) exposure to sunlight (f) contaminated food (g) Others, please specify……

13. What are the symptoms of malaria (do not read out options, you may probe: are there are any more symptoms?): a) fever (b) headache (c) joint pain (d) generalised body weakness (e) generalised body pain (f) loss of appetite (g) vomiting (h) diarrhoea (i) anaemia (j) convulsion (k) others, please specify……. (Multiple response)

14. ANC education and counselling should include consequences of malaria in pregnancy to both mother and child: a) Yes b) No (c) I don’t know

15. Malaria parasite in the blood of pregnant women interferes with transfer of nutrients (food) to the baby: a) Yes b) No (c) I don’t know

16. Use of long-lasting insecticidal nets (LLINs) do not reduce malaria transmission a) Yes b) No (c) I don’t know

17. What do you understand by intermittent preventive treatment of malaria during pregnancy? ____________________ (*Hint: Intermittent Preventive Treatment of malaria during pregnancy is the administration of a dose of 3 white tablets of SP to a pregnant woman*)

18. What is the current medicine recommended by National Malaria Elimination Programme for malaria prevention during pregnancy: a) Sulphadoxine-Pyrimethamine b) Pyrimethamine c) Proguanil d) Chloroquine e) Artesunate f) No medicine/medication g) others (please specify)

19. Intermittent preventive treatment can be given to (choose one best option): a) Men b). Pregnant Women c). Aged People d). Infant

20. Intermittent preventive treatment with SP during pregnancy should be commenced (*choose all that apply*) : a) after 13 weeks b) at quickening (from 16 weeks onwards) c) Mid-half of pregnancy (20 weeks) d) First trimester e) Third trimester f) 2^nd^ trimester

21. Sulphadoxine-Pyrimethamine (SP) should be given to women in 1^st^ trimester: a) Yes b) No c) I don’t know

22. Pregnant women should be given SP to take at home: a) Yes b) No c) I don’t know

23. SP should be given to a woman who had received recent treatment with SP less than 1 month ago: a) Yes b) No c) I don’t know

24.SP should be given in 2-doses to HIV negative pregnant women? a) Yes b) No c) I don’t know

25. SP should be given only to HIV negative pregnant women not receiving cotrimoxazole? a) Yes b) No c) I don’t know

**SECTION D:** **KNOWLEDGE OF HEALTH CARE PROVIDERS ON MALARIA CASE MANAGEMENT**

26. How do you ascertain someone has malaria?

a. Fever and or any other symptoms and signs only 🞏 Yes 🞏 No

b) Fever and or any other symptoms and signs with parasitological confirmation of malaria 🞏 Yes 🞏 No

27. What is the currently recommended first-line treatment for uncomplicated malaria in Nigeria?

Artemether-Lumefantrine (e.g. Coartem) 🞏

Artesunate-Amodiaquine 🞏

Quinine tablets 🞏

Dihydroartemisinin – piperaquine 🞏

Quinine injection 🞏

Artemether injection 🞏

Artesunate 🞏

Amodiaquine 🞏

Tablet Chloroquine 🞏

Injection Chloroquine 🞏

Sulphadoxine-pyrimethamine 🞏

Halofantrine 🞏

Others, please specify ____________________

28. Please fill as appropriate if you were to give a patient Tab Artemether-Lumefantrine 20/120mg formulation

| **Age** | **Dosing regimen** | | | |
| --- | --- | --- | --- | --- |
|  | **Number of tablets given each at time** | **Number of times per day** | **For how many days?** | **Time interval between 1^st^ and 2^nd^ dose (hrs)** |
| 6mths – 3yrs |  |  |  |  |
| 4yrs – 7yrs |  |  |  |  |
| 8yrs – 13yrs |  |  |  |  |
| ≥ 14ys |  |  |  |  |

29. Please fill as appropriate if you were to give a patient Tab Artesunate-Amodiaquine

| **Age** | **Dosing regimen** | | | |
| --- | --- | --- | --- | --- |
|  | **Number of tablets given each at time** | **Number of times per day** | **For how many days?** | **Time interval between 1^st^ and 2^nd^ dose (hrs)** |
| 2mths – 11months |  |  |  |  |
| ≥1yr – 5yrs |  |  |  |  |
| 6 – 13yrs |  |  |  |  |
| ≥ 14ys |  |  |  |  |

30) What is the main method of storage of antimalarials in the health facility (might have to ask the pharmacist/pharmacy technician)?

No special arrangement 🞏

Cold boxes 🞏

Cool environment 🞏

Away from sunlight/heat 🞏

Away from moisture 🞏

Moisture-proof envelopes 🞏

Others (Specify) ____________________

31) What are the conditions that need to be considered for RDT storage (might have to ask the laboratory scientist/technician)? (Tick all that apply)

Temperature 🞏

Humidity 🞏

Others, please specify ____________________

32) What is the main method of storage of RDT kits in the health facility?

No special arrangement 🞏

Cold boxes 🞏

Cool environment 🞏

Away from sunlight/heat 🞏

Moisture-proof envelopes 🞏

Others (Specify) ____________________

**SECTION E: Healthcare workers practices of malaria treatment**

33). Do you have national diagnosis and treatment guideline (sight before you tick ‘Yes’)?

🞏 Yes 🞏 No I Don’t Know 🞏

(if No or I Don’t Know, skip Q34)

34) Do you use the national diagnosis and treatment guideline for malaria management?

🞏 Yes 🞏 No I Don’t Know 🞏

35) Have you had any supervisory visit by officers of the State ministry of health in the last 6 months?

🞏 Yes 🞏 No I Don’t Know 🞏

36). Do you have posters for IPT displayed at your health facility (sight before you tick ‘Yes’) : a) Yes b) No I Don’t Know 🞏

37). How do you make the diagnosis of malaria (choose all that apply)? a) health worker recognition of signs b) Confirmation of presence of parasites using microscopy c) Confirmation of presence of parasites using malaria rapid diagnostic test d) patient self-recognition of symptoms

38). What is the best way to treat a patient confirmed to have malaria? a) administration of antimalarial medicines b) no treatment c) diet modification e) religious healing f) others, please specify, ………

39). Which medicine do you administer (or prescribe if none is available at the health facility) for the treatment of malaria? Tick all that apply a) Oral Artesunate monotherapy b) Chloroquine tablets c) Chloroquine injection d) Antibiotics e) Sulphadoxine-Pyrimethamine (SP) f) Quinine tablets g) Quinine injectable h) Artemisinin-based combination therapy (ACT) i) Herbal preparations (j) Others, please specify……

40). Which antimalarial (s) formulation do you usually administer for uncomplicated malaria? (Tick all that apply) a) Intramuscular Injection b) Tablets c) Intravenous infusion (IV) d) suppositories e) others, please specify….

41). Which type of antimalarial (s) do you administer for the treatment of uncomplicated malaria during pregnancy in the first trimester. (Tick all that apply) a) Quinine tablets b) ACT c) Sulphadoxine-pyrimethamine d) Chloroquine tablets e) Chloroquine injection f) Proguanil g) Amodiaquine h) Oral Artesunate monotherapy i) Injectable Artesunate j) Artemether injection (k) Quinine injection l) others, please specify…..

42). Which antimalarial do you use for the treatment of uncomplicated malaria during pregnancy in the second and third trimesters a) Quinine tablets b) ACT c) Sulphadoxine-pyrimethamine d) Chloroquine tablets e) Chloroquine injection f) Proguanil g) Amodiaquine h) Oral Artesunate monotherapy i) Injectable Artesunate j) Artemether injection (k) Quinine injection l) others, please specify…..

43). Do you inform pregnant women when next they are due for the next dose of IPTp-SP: a) Yes b) No c) I don’t know

44). Do you advise pregnant women to come to the clinic if there are reactions to anti-malarial medicines: a) Yes b) No c) I don’t know

45). Do you offer another antimalarial medicine when you perceive a patient does not get better with any of the 1^st^ treatment? 🞏 Yes 🞏 No 🞏 I don’t know

46). What antimalarial medicines do you prescribe when you perceive a patient does not get better with any of the 1^st^ treatment (Tick all that apply)

Quinine tablets 🞏

Dihydroartemisinin – piperaquine 🞏

Quinine injection 🞏

Artemether Injection 🞏

Injectable Artesunate 🞏

Amodiaquine 🞏

Chloroquine injection 🞏

Sulphadoxine-pyrimethamine 🞏

Halofantrine 🞏

Rectal artesunate 🞏

Others, please specify) ____________________

47) In your opinion, what factors influence the type of antimalarial medicines prescribed in the facility (Tick all that apply)?

Necessity to make profit 🞏

Request by patients 🞏

Understanding of patients’ preferences 🞏

Available medicines 🞏

Existing treatment policy/guidelines 🞏

Medicine promoted by manufacturers 🞏

Don’t know 🞏

Others (please specify) ____________________

48) What is the main reason for selecting artemisinin derivatives?

Therapeutic efficacy (higher parasite clearance rate) 🞏

Lower pill burden (fewer tablets) 🞏

Few adverse effects 🞏

Medicine cost (lowest price) 🞏

Name of medicines 🞏

Patient's request 🞏

National guideline recommendation 🞏

Don’t know 🞏

49). What is the main source of ACT in your health facility?

Government 🞏

Bought from a pharmacy 🞏

Donated by an NGO 🞏

Not sure 🞏

Don’t know 🞏

Others, please specify) ____________________

50. These features might make you refer a patient to higher level of health care. Please tick as appropriate.

| Fever/history of fever in the last 24 hours | 🞎Yes | 🞎No |
| --- | --- | --- |
| Headache | 🞎Yes | 🞎No |
| Loss of appetite | 🞎Yes | 🞎No |
| Joint pains | 🞎Yes | 🞎No |
| General weakness | 🞎Yes | 🞎No |
| Nausea/Vomiting | 🞎Yes | 🞎No |
| Prostration *(i.e. generalised weakness or inability to sit, stand or walk without support)* | 🞎Yes | 🞎No |
| Impaired consciousness *(confusion or drowsiness or coma)* | 🞎Yes | 🞎No |
| Respiratory distress *(difficulty in breathing, fast deep breath)* | 🞎Yes | 🞎No |
| Convulsion (s) | 🞎Yes | 🞎No |
| Circulatory collapse *(shock)* | 🞎Yes | 🞎No |
| Pulmonary oedema *(respiratory distress/radiology)* | 🞎Yes | 🞎No |
| Abnormal bleeding *(disseminated intravascular coagulopathy)* | 🞎Yes | 🞎No |
| Jaundice *(yellow discoloration of the eyes)* | 🞎Yes | 🞎No |
| Haemoglobinuria *(Coca-Cola coloured urine)* | 🞎Yes | 🞎No |
| Severe anaemia *(Hb <5 gm/dl)* | 🞎Yes | 🞎No |
| Hypoglycaemia | 🞎Yes | 🞎No |
| Renal Failure *(not making enough urine)* | 🞎Yes | 🞎No |
| Persistent/excessive vomiting | 🞎Yes | 🞎No |
| Inability to drink or breastfeed | 🞎Yes | 🞎No |

51. Have you had stock out of any of these antimalarials consecutively for 7days in the past one month (Tick as appropriate)?

| **S/N** | **Medicine** | **Yes** | **No** | **Don’t normally stock** |
| --- | --- | --- | --- | --- |
| 1 | ACT |  |  |  |
| 2 | Sulphadoxine-pyrimethamine |  |  |  |
| 3 | Tabs Quinine |  |  |  |
| 4 | Quinine Injection |  |  |  |
| 5 | Tabs Chloroquine |  |  |  |
| 6 | Chloroquine injection |  |  |  |
| 7 | Antibiotics |  |  |  |
| 8 | Injectable Artesunate |  |  |  |
|  | Others, please specify………. |  |  |  |

**SECTION E: Data management (information to be obtained from record officer). Data collector should observe each register)**

52. Please fill as appropriate for each tool

| 1 | Daily attendance register | 🞎 Available, correctly filled up till date | 🞎 Available, incorrectly filled | | 🞎 Adequate | 🞎 Available, not used | 🞎 Not available |
| --- | --- | --- | --- | --- | --- | --- | --- |
| 2 | Daily Outpatient Department register | 🞎 Available, correctly filled up till date | | 🞎 Available, incorrectly filled | 🞎 Adequate | 🞎 Available, not used | 🞎 Not available |
| 3 | Daily ANC register | 🞎 Available, correctly filled up till date | | 🞎 Available, incorrectly filled | 🞎 Adequate | 🞎 Available, not used | 🞎 Not available |
| 4 | Immunisation register | 🞎 Available, correctly filled up till date | | 🞎 Available, incorrectly filled | 🞎 Adequate | 🞎 Available, not used | 🞎 Not available |
| 5 | In-patient care daily register | 🞎 Available, correctly filled up till date | | 🞎 Available, incorrectly filled | 🞎 Adequate | 🞎 Available, not used | 🞎 Not available |
| 6 | Monthly summary form | 🞎 Available, correctly filled up till date | | 🞎 Available, incorrectly filled | 🞎 Adequate | 🞎 Available, not used | 🞎 Not available |

**Hint:** Adequate tool refers to tool that will be sufficient to capture data for the next 3 months based on average monthly patient load

57. How often do you use the data for decision making e.g., number of cases of malaria seen over a period of time (for officer -in-charge of health facility)?

a) regularly b) rarely c) occasionally d) never did

| RECORD THE TIME THE INTERVIEW ENDED | \|  \|  \|  \|  \| . \|  \|  \|  \|  \| \| --- \| --- \| --- \| --- \| --- \| --- \| --- \| --- \| --- \| \|  \|  \|  \|  \| . \|  \|  \|  \|  \| |
| --- | --- | --- | --- | --- | --- | --- | --- | --- | --- | --- | --- | --- | --- | --- | --- | --- | --- | --- | --- |

Thank you
